# Increased *Trypanosoma cruzi* Growth during Infection of Macrophages Cultured on Collagen I Matrix

**DOI:** 10.3390/life13041063

**Published:** 2023-04-21

**Authors:** Jorgete Logullo, Israel Diniz-Lima, Juliana Dutra B. Rocha, Suzana Cortê-Real, Elias Barbosa da Silva-Júnior, Joyce Cristina Guimarães-de-Oliveira, Alexandre Morrot, Leonardo Marques da Fonseca, Leonardo Freire-de-Lima, Debora Decote-Ricardo, Celio Geraldo Freire-de-Lima

**Affiliations:** 1 Instituto de Biofísica Carlos Chagas Filho, Universidade Federal do Rio de Janeiro, Rio de Janeiro 21941-901, RJ, Brazil; 2 Laboratório de Biologia Estrural, Instituto Oswaldo Cruz, FIOCRUZ, Rio de Janeiro 21040-360, RJ, Brazil; 3 Laboratório de Imunoparasitogia, Instituto Oswaldo Cruz, FIOCRUZ, Rio de Janeiro 21040-360, RJ, Brazil; 4 Faculdade de Medicina, Universidade Federal do Rio de Janeiro, Rio de Janeiro 21941-901, RJ, Brazil; 5 Instituto de Veterinária, Universidade Federal Rural do Rio de Janeiro, Seropédica 23890-000, RJ, Brazil

**Keywords:** extracellular matrix, macrophage, *Trypanosoma cruzi*, collagen I, 3D matrix

## Abstract

The interactions between cell and cellular matrix confers plasticity to each body tissue, influencing the cellular migratory capacity. Macrophages rely on motility to promote their physiological function. These phagocytes are determinant for the control of invasive infections, and their immunological role largely depends on their ability to migrate and adhere to tissue. Therefore, they interact with the components of the extracellular matrix through their adhesion receptors, conferring morphological modifications that change their shape during migration. Nevertheless, the need to use in vitro cell growth models with the conditioning of three-dimensional synthetic matrices to mimic the dynamics of cell-matrix interaction has been increasingly studied. This becomes more important to effectively understand the changes occurring in phagocyte morphology in the context of infection progression, such as in Chagas disease. This disease is caused by the intracellular pathogen *Trypanosoma cruzi*, capable of infecting macrophages, determinant cells in the anti-trypanosomatid immunity. In the present study, we sought to understand how an in vitro extracellular matrix model interferes with *T. cruzi* infection in macrophages. Using different time intervals and parasite ratios, we evaluated the cell morphology and parasite replication rate in the presence of 3D collagen I matrix. Nevertheless, microscopy techniques such as scanning electron microscopy were crucial to trace macrophage-matrix interactions. In the present work, we demonstrated for the first time that the macrophage-matrix interaction favors *T. cruzi* in vitro replication and the release of anti-inflammatory cytokines during macrophage infection, in addition to drastically altering the morphology of the macrophages and promoting the formation of migratory macrophages.

## 1. Introduction

The extracellular matrix (ECM) is composed of proteins and polysaccharides that are secreted by the different cell types that make up the tissues. The various tissues are characterized not only by the constituent cell types but also by the chemical composition, structure, and mechanics of their ECMs [1]. Such properties modulate various cellular functions. Both in vitro and in vivo, cells and their respective ECMs are in constant communication, where the recognition of the extracellular environment by the receptors and co-receptors present on cell surfaces trigger functional modifications [2,3,4].

Collagen is comprised of fibrous proteins of predominant occurrence in the extracellular environment and are involved not only in the supramolecular structure of the ECMs but also in the modulation of several biological processes such as adhesion, migration, growth, differentiation, and tissue repair [5,6].

Collagen I is the most abundant type of collagen in mammalian tissues and, therefore, the most easily obtainable in large quantity [7], ECMs constituted by collagen I and structured in three-dimensional (3D) are the most studied models, both with respect to the mechanics presented by collagen I and their interaction with eukaryotic cells [8,9].

The most used materials in mammalian in vitro cell culture are composed of rigid and planar substrates made of glass or plastics and are subjected to special treatments. In recent decades it was observed a growing interest in the culture of mammalian cells, particularly human cells, in 3D frameworks in the scientific literature. That means the use of cell culture models with rigid or flexible/floating substrates structured in 3D scaffolds [10]. This tendency is mainly due to the fact that this type of crop provides a mechanochemical environment closer to that observed in vivo [11].

Macrophages are very active cells with amoeboid movement and variable morphology, depending on their functional state, location, and chromatin shape [12,13,14]. It plays an important role in the removal of cell debris and altered extracellular material. They influence cell activity and homeostasis in various tissues and are antigen-presenting cells, pivotal for the initial recognition and removal of senescent cells [15]. They also provide a frontal line of defense against invading antigens and newly formed tumor cells [16,17].

Macrophages interact with other cells and the ECM in various situations during their ontogeny and differentiation, as well as in response to appropriate physiological stimuli. Its ability to interact with extracellular matrix molecules, such as collagen, laminin, and fibronectin, is a determining factor for the development of its unique functions [18]. These interactions allow the cells to adhere and spread over the matrix and occur via specific cell membrane receptors, including some integrins [19].

Macrophages are part of the innate immune system, recognizing, phagocytosing, and destroying many potential pathogens, including bacteria, protozoa, and fungi [20]. Phagocytosis begins with the spreading, characterized by the alteration of the form and redistribution of the cytoplasmic organelles. Changes in cell shape increase the contact area of the plasma membrane of the phagocyte with the substrate. During adhesion, the activation of membrane receptors for adhesion molecules occurs, causing the rearrangement of the cellular cytoskeleton [21,22].

*Trypanosoma cruzi* is an obligatory intracellular protozoan parasite, and it is the etiological agent of Chagas disease, which is endemic in the Americas. In addition, a wide spectrum of mammalian cells can be infected by *T. cruzi*, including macrophages [23]. Besides acting as target cells for the multiplication of parasites, macrophages also act as effector cells in the elimination of intracellular parasites [24]. Although the study of macrophages in two-dimensional (2D) rigid surface material has already been well evaluated [25], the mechanisms involved in macrophage/matrix interaction are still poorly documented.

Although ECM constituents are still little studied in the context of cellular modulation, they seem to be crucial for cell modulation in immunopathological processes. Interestingly, the migration of macrophages in the context of the presence of ECM is modulated by biomechanical factors and cell adhesion, which are different according to the matrix composition [26]. In addition, the presence of ECM strongly influences the resident macrophage phenotype, as macrophages conditioned with ECM clearly become more regulatory in phenotype, which is the opposite of what is observed for macrophages cultured in the absence of matrix [27]. Furthermore, the different physiological origin of the ECM differently activates macrophages in culture, again associated with the difference in structural composition [27]. In this sense, matrices with fibrin in their composition induce a more regulatory profile in macrophages, associated with the initiation of tissue repair mechanisms mobilized by alternatively activated macrophages [28]. Furthermore, collagen-based matrices also elicit the same regulatory effect, demonstrating that this phenomenon is not exclusive to the presence of fibrin. Nevertheless, a tighter interaction between the components of the ECM, increasing its density, is associated with greater macrophage migration, adhesion, and activation of the inflammatory response due to tumor necrosis factor-alpha (TNF-α) secretion [29]. Therefore, ECMs are cellular scaffolds that vary in composition and physical structure, strongly influencing the activation of macrophages, being crucial to tissue repair and pathogen clearance.

It is known that macrophages interact with the collagen type I in the matrix through the VLA-1 and VLA-2 surface receptors [30,31,32]. Thus, the aim of this work was to evaluate the role of macrophage/collagen in the 3D collagen type I matrix (3D matrix) interaction in the context of *T. cruzi* infection in vitro.

## 2. Materials and Methods

### 2.1. Animals

Male BALB/c mice, aged 6–8 weeks, were obtained from the Center for Creation of Laboratory Animals (CECAL), Fiocruz-RJ, and kept in microisolators, with food and water *ad libitum*. All experimental procedures were carried out in accordance with the rules for the use of laboratory animals established by the UFRJ Ethics Committee. (CEUA No.: 079/19). Male Wistar rats, aged 8–10 weeks, were obtained from an Animal Care Facility in the Health Science Center of the Federal University of Rio de Janeiro, Rio de Janeiro, Brazil. All experimental procedures were carried out in accordance with the rules for the use of laboratory animals established by the UFRJ Ethics Committee. (CEUA No.: 045/17).

### 2.2. Parasites

*T. cruzi* clone Dm28c was used in all experiments. The epimastigotes were cultivated, through successive passages, in BHI medium (Difco/Becton & Dickinson Laboratories, Detroit, MI, USA) supplemented with hemin (25 mg/L, Sigma Chemical Co., St. Louis, MO, USA), folic acid (20 mg/L, Sigma Chemical Co.) and 10% fetal bovine serum (FBS) inactivated at 56 °C (FBS, Gibco-Invitrogen, Grand Island, NY, USA), and kept in a BOD incubator at 27 °C. The metacyclic forms of *T. cruzi* clone Dm28c were differentiated in vitro in TAU-P (Triatomine Artificial Urine) medium (190 mM NaCl; 8 mM sodium phosphate buffer (PBS), pH 6.0; 17 mM KCl; 2 mM CaCl_2_; 2 mM MgCl_2_; 0.035% NaHCO_3_, with the addition of 10 mM L-proline; all from Sigma Chemical Co.) according to the protocol described by Contreras and collaborators (1985) [33].

### 2.3. Collagen I Source

Collagen I used in the 2D and 3D models was isolated from the tail of Wistar rats through the following process: the tails were first washed with detergent and immersed in acetone for 5 min, having been left to rest in ethanol until the day of the extraction. With the help of tweezers and scissors, each vertebra of the tail was broken to remove the collagen fibers. The removed fibers were placed in a 1% acetic acid solution for 96 h at 4 °C for solubilization. Subsequently, the solution was subjected to ultracentrifugation at 30,000× *g* (BECKMAN COULTER, OPTIMA L-90) for 45 min at 4 °C to discard non-solubilized fibers. The supernatant was conserved and dialyzed in 1% chloroform for 1 h to sterilize the solution. Then, the solution was dialyzed in sterile 0.02 N acetic acid for 3 days, changing the solution daily. At the end of dialysis, the product was stored at 4 °C. Protein concentration was determined by the dry weight method [34].

### 2.4. Macrophage Harvesting

Resident peritoneal macrophages were obtained from BALB/c mice by washing the peritoneal cavity of these animals with 5 mL of DMEM supplemented with 2 mM L-glutamine, 50 μM β-mercaptoethanol, 1 mM pyruvate, 10 μg/mL of gentamicin and 0.1 mM non-essential amino acids (all from Gibco-Invitrogen) and without FBS. Cells were counted in a Neubauer chamber using Trypan Blue dye (Sigma) for cell viability analysis, adjusted to a concentration of 6 × 10^5^ cells/mL in DMEM supplemented with 10% FBS and 1 mL of this suspension was distributed in triplicates in 24-well plates for cell culture with or without 2D biofilm or 3D matrix layer. The plates were incubated at 5% CO_2_ and 37 °C for 3 h for macrophages to adhere.

### 2.5. Interaction Models In Vitro

Three different models of in vitro macrophage interaction were conducted. One consisting in direct culturing the polystyrene surface of 24-well plates (Corning, New York, NY, USA) with 1 mL of the macrophage suspension in DMEM (Dulbecco’s Modified Eagle’s Medium) (Gibco-Invitrogen) at 6 × 10^5^ cells/well. The other two protocols consisted in culturing the macrophages on different surfaces, 2D collagen type I biofilm (2D biofilm) and 3D collagen type I matrix (3D matrix).

#### 2.5.1. D Collagen Type I Biofilm

The 2D biofilm model of macrophage interaction with collagen I was made by coating the surface of 24-well plates with 300 μL of collagen I solution at 50 μg/mL in DMEM, which were incubated at 5% CO_2_ and 37 °C for 1 h. Then, the excess solution was aspirated, and 1 mL of the macrophage suspension in DMEM was added to the wells (6 × 10^5^ cells/well) [35].

#### 2.5.2. D Collagen Type I Matrix

The 3D matrix model was made by using collagen I at a concentration of 1.5 mg/mL. For the matrix construction, the concentrated collagen I solution was diluted according to the following protocol: an aliquot of 480 μL of the 5-fold concentrated DMEM was diluted in a 50 mL tube with 5120 μL of 1× DMEM, 189 μL of NaOH, and 2400 μL of collagen I 5 mg/mL so that the final concentration of the matrix corresponded to 1.5 mg/mL and pH 7.0. The entire process was carried out on the ice to prevent gelatinization from occurring before the ideal time. The 300 µL of collagen I solution at 1.5 mg/mL added to the wells of the 24-well plates, and the plates were incubated at 5% CO_2_ and 37 °C for 1 h to complete the gelatinization process [35]. Then, 1 mL of the macrophage suspension was added at 6 × 10^5^ cells/mL.

### 2.6. Parasitic Burden Assay

Murine macrophages obtained, as described in Section 2.4, were infected overnight (6 × 10^5^ adherent cells/well in 24-well culture vessels) with chemically induced metacyclic forms of *T. cruzi* clone Dm28c at a 1:1 and 3:1 parasite:cell ratio in 1 mL of complete culture medium containing 10% FCS at 37 °C. On the following day (day 1), monolayers were extensively washed to remove extracellular parasites and cultured with a complete culture medium containing 1% Nutridoma instead of FCS.

The fold change in parasite number was evaluated after seven and ten days in culture by counting the free trypomastigotes and amastigotes in the supernatants in a Neubauer chamber.

In some experiments, resident peritoneal macrophages of normal BALB/c mice cultured or not on a layer of 2D biofilm at a concentration of 50 μg/mL or 3D matrix at a concentration of 1.5 mg/mL were stimulated with 10 ng/mL of lipopolysaccharide (LPS from *Salmonella typhimurium*, Sigma Chemical Co.) and 40 U/mL of interferon-gamma (IFN-γ) for 24 h. The supernatants of these cultures were collected and stored at −20 °C for cytokine quantification.

The macrophages on the collagen I models were subjected to an enzymatic digestion process with a 1 mg/mL collagenase solution 300 μL/well (Type IA, Sigma Chemical, St. Louis, MO, USA). The plates were incubated at 5% CO_2_ and 37 °C for 20 min, and then the cells obtained after this enzymatic digestion were adjusted to 1 × 10^5^ cells/200 μL and cytocentrifuged at 6000 rpm for 3 min in a centrifuge (CT-2000 CIENTEC). After centrifugation, cells were stained using the Instant Prov kit (LABORCLIN) and then observed under an optical microscope (ZEISS, Oberkochen, Germany, 100X).

### 2.7. Determination of Cytokines

The concentrations of transforming growth factor beta (TGF-β) and TNF-α in the supernatant, obtained from cultures of infected macrophages cultured on 2D biofilm or 3D matrix, were quantified after 48 h of incubation by sandwich immunoassay (ELISA) according to methodology recommended by the manufacturer (R&D). The optical density was obtained from a plate spectrophotometer (VERSAMAX MICROPLATES Reader Molecular Devices, San Jose, CA, USA). The concentrations of cytokines were calculated from a standard curve of recombinant cytokines.

### 2.8. Determination of Nitric Oxide

Nitric oxide (NO) produced by peritoneal macrophages cultured on 2D biofilm, 3D matrix, or plates was quantified by the presence of nitrite accumulated in the supernatant of cultures using the Griess colorimetric method [36].

### 2.9. Scanning Electron Microscopy (SEM)

Normal or *T. cruzi*-infected macrophages were fixed with 2.5% glutaraldehyde in 0.1 M cacodylate buffer (pH 7.2) for 1 h and then washed overnight with PBS. After fixation, trypomastigotes in the 3D matrix were postfixed for 30 min in a solution containing 1% OsO_4_, 1.25% potassium ferrocyanide, and 5 mM CaCl_2_ in 0.1 cacodylate buffer, washed in the same buffer, and then dehydrated in an ethanol series from 30% to 100%. Finally, samples were critical point dried, coated with a thin gold layer in a gold sputtering system (Oerlikon, Balzers), and observed in an FEI-Quanta scanning electron microscope [37].

### 2.10. Statistical Analysis

Results were expressed as mean ± standard error (SE). Statistical analyzes were performed using Prism 6 (Sigma) for Windows using the “One-way ANOVA” method.

## 3. Results

### 3.1. Infected Macrophages Grown in a 3D Environment Release More Trypomastigotes Forms

Our first hypothesis was that the replication rates of *T. cruzi* in murine macrophages after in vitro infection would be altered with different chemical or mechanical matrix compositions. To address that, we cultured peritoneal macrophages and infected them with *T. cruzi* on a layer of collagen type I in a 2D biofilm form or in a 3D matrix layer, or directly on the polystyrene surface of 24 well plates.

**Figure 1:**
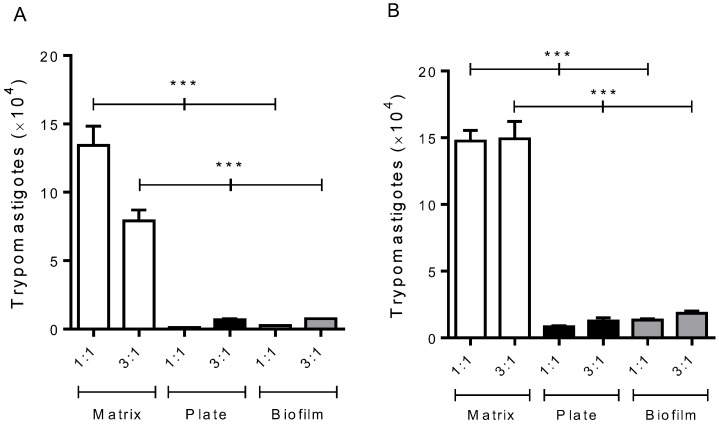
The presence of a 3D matrix in vitro induces a higher trypomastigote release from *T. cruzi*-infected macrophages. Peritoneal macrophages from BALB/c mice (6 × 10^5^) were cultured on the 3D matrix or 2D biofilm or directly on the wells of 24-well plates. After 3 h of macrophage adhesion to different surfaces, cells were infected with metacyclic forms of *T. cruzi* clone Dm28 at parasite:cell ratios of 1:1 (6 × 10^5^) and 3:1 (18 × 10^5^). Then, 24 h later, the infected monolayers were extensively washed to remove extracellular parasites. After seven and ten days of infection, the trypomastigote forms released in the culture supernatants were counted using a Neubauer chamber. Each bar represents the mean ± SE of triplicates from a representative experiment. The experiments were repeated three times, and similar results were obtained. Figures: (**A**) (seven days post-infection), (**B**) (ten days post-infection). *** *p* < 0.001.

We observed a marked increase in *T. cruzi* replication occurring in the 3D matrix situation after 7 and 10 days of culture (Figure 1A,B), respectively. This result suggests that macrophage infection in the 3D matrix environment behaves differently from the one in the polystyrene plate and in 2D biofilm surfaces.

The increased parasite replication in the 3D matrix system may be associated with changes in cell shape, which represents the area of contact between the macrophage plasma membrane and the substrate (3D matrix), which would favor the infection of the cell by the *T. cruzi*. Infected macrophages cultured on 2D biofilm released levels of parasites similar to the ones observed for macrophages cultured directly on the plate, suggesting that the effect is mechanical rather than chemical (Figure 1).

### 3.2. Infected Macrophages Cultured on a 3D Matrix System Release More Amastigotes into the Extracellular Environment

Based on previous studies showing that trypomastigotes forms from cell culture differentiate into amastigotes in culture medium at 37 °C in vitro [38,39], we decided to investigate whether this differentiation was influenced by the different collagen I model employed in this study. Since there was no difference in the parasitic burden after only 7 days of infection, we only analyzed parasite differentiation after 10 days. We then evaluated the number of amastigotes in the supernatants of infected macrophages cultured for 10 days on a layer of 2D biofilm, on a 3D matrix, and directly on the polystyrene surface of 24-well plates.

**Figure 2:**
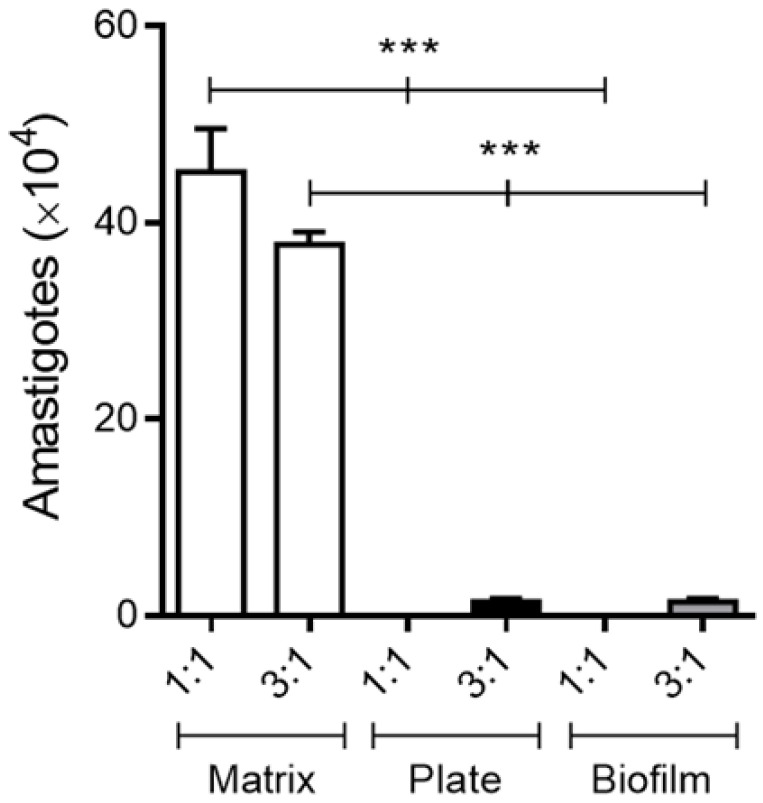
The presence of a 3D matrix in vitro induces a higher amastigote release from *T. cruzi*-infected macrophages. Peritoneal macrophages from BALB/c mice (6 × 10^5^) were cultured on the 3D matrix or 2D biofilm or directly on the wells of 24-well plates. Macrophage monolayers were infected after 3 h of adhesion with metacyclic forms of *T. cruzi* clone Dm28c at parasite:cell ratios of 1:1 (6 × 10^5^) and 3:1 (18 × 10^5^). Then, 24 h later, the infected monolayers were extensively washed to remove extracellular parasites. After ten days of incubation, the amastigote forms released in the culture supernatants were counted in a Neubauer chamber. The results (means ± SE of triplicates) presented are representative of three experiments performed separately. *** *p* < 0.001.

We observed a difference in the number of extracellular amastigote forms in the supernatants of cultures of infected macrophages cultured on a 3D matrix when compared to the other variants. We can suggest that the smaller number of trypomastigote forms observed on the seventh day of incubation in the monolayers of macrophages cultured on a 3D matrix layer (higher concentration ratio of infection) was related to an early release of trypomastigotes and subsequent transformation into amastigotes in vitro (Figure 2).

### 3.3. Culture of Infected Macrophages on the 3D Matrix Induces the Early Release of Trypomastigote Forms

**Figure 3:**
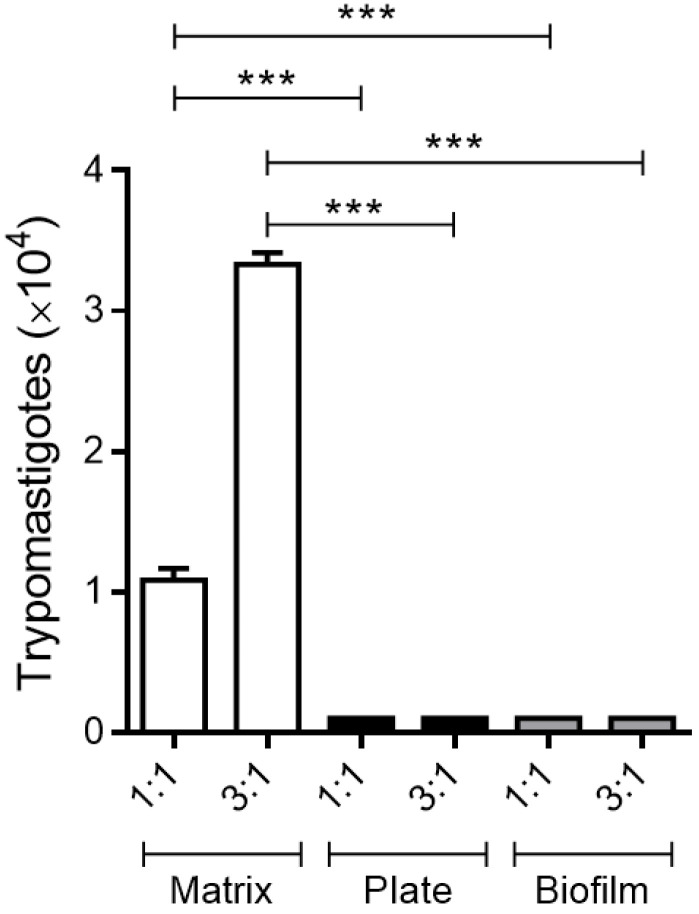
The presence of a 3D matrix in vitro induces early trypomastigote release from *T. cruzi*-infected macrophages. Peritoneal macrophages from BALB/c mice (6 × 10^5^) were cultured on the 3D matrix or directly on the wells of 24-well plates. Cell cultures were infected after 3 h of adhesion with metacyclic forms of *T. cruzi* clone Dm28c in proportions of 1:1 (6 × 10^5^) and 3:1 (18 × 10^5^). 24 h later, the infected monolayers were extensively washed to remove extracellular parasites. After 96 h of infection, trypomastigote forms released in the culture supernatants were counted using a Neubauer chamber. The results (means ± SE of triplicates) presented are representative of three experiments performed separately. *** *p* < 0.001.

In order to verify if the results presented in Figure 1 and Figure 2 were indeed related to the early release of trypomastigote forms by macrophages distributed on a 3D matrix, the free parasite counts in the supernatant of cells cultured in two experimental conditions were performed after 96 h of incubation at 37 °C. Even after only 96 h of culture, it was already possible, in the 3D model, to count trypomastigote forms in the supernatants (Figure 3).

**Figure 4:**
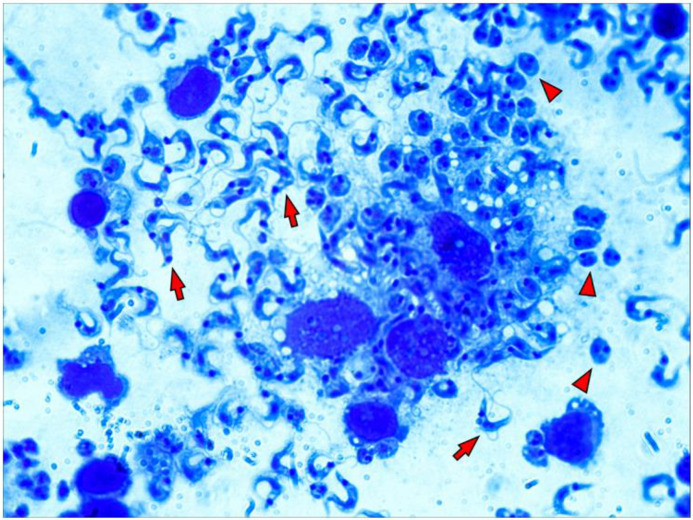
Macrophages are intensely parasitized early in vitro in the 3D matrix. Peritoneal macrophages from BALB/c mice (6 × 10^5^/well) were distributed into wells of 24-well plates covered with 3D matrix and allowed to adhere for 3 h at 5% CO_2_ and 37 °C. Cells were infected overnight with metacyclic forms of *T. cruzi* clone Dm28c in a 3:1 proportion (18 × 10^5^). After this incubation, the wells were washed to remove non-internalized parasites. Enzymatic digestion with collagenase was performed 96 h post-infection, and the suspension obtained was used in the preparation of slides by a cytocentrifuge. The slides were fixed and stained using the Instant Prov kit (LABORCLIN) and then observed under an optical microscope (ZEISS, 100X). Trypomastogotes are indicated with red arrows, and amastigotes are indicated with red arrowheads.

As already mentioned, amastigote forms divide intensely in the cytoplasm of the host cell, then differentiate into trypomastigotes and rupture the cell membrane. For a more detailed observation of the macrophages cultured on a 3D matrix layer infected by *T. cruzi*, we subjected the culture to an enzymatic digestion process with collagenase I after 96 h of infection. The suspensions obtained were used in the preparation of microscopy slides using a cytocentrifuge. The evaluation of the slides under optical microscopy showed intensely parasitized macrophages and trypomastigote forms disrupting the host cell membrane (Figure 4).

### 3.4. Detection of TNF-α and TGF-β

**Figure 5:**
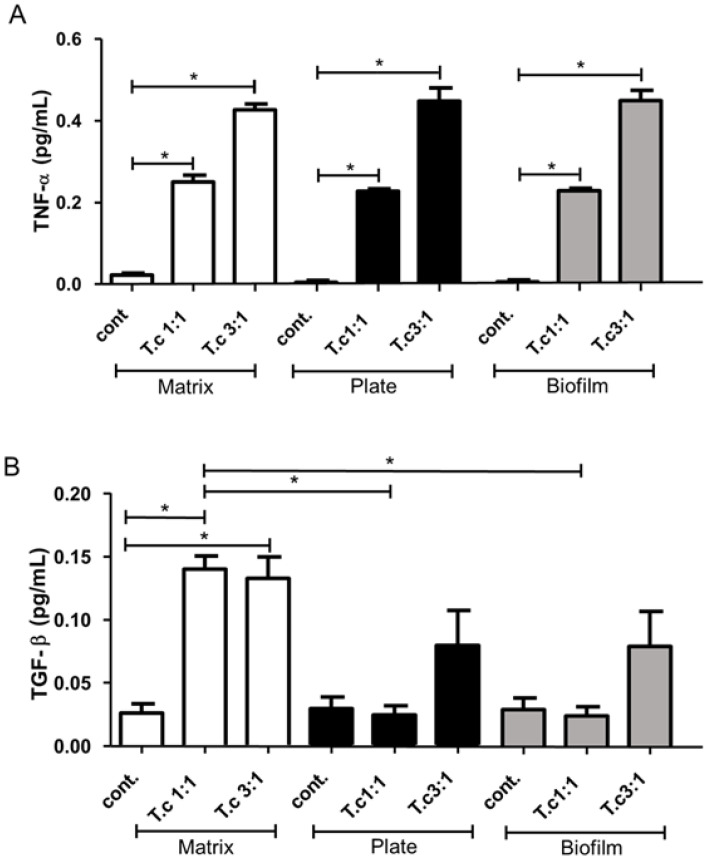
Higher parasite:cell ratio induces macrophages to produce more TNF-α in vitro, and macrophages grown in 3D matrix produce more TGF-β Peritoneal macrophages from BALB/c mice (6 × 10^5^) were cultured on the 2D biofilm, 3D matrix or on the polystyrene surface of the 24-well plates. Cell cultures were infected after 3 h of adhesion with metacyclic forms of *T. cruzi* in proportions of 1:1 (6 × 10^5^) and 3:1 (18 × 10^5^) and incubated overnight at 5% CO_2_ and 37 °C. After 48 h of culture, the production of TNF-α (**A**) and TGF-β (**B**) was evaluated in the supernatants. The results (means ± SE of triplicates) presented are representative of three experiments performed separately. * *p* < 0.05.

To evaluate the cytokine production by macrophages, a control group of non-infected cells was introduced to compare the basal levels between infected groups and collagen I models. It is known that the proinflammatory cytokine TNF-α produced by macrophages is essential in the immune response against *T. cruzi* [40]. The cytokine analysis shows no difference in TNF-α production between macrophages cultured and infected on a 3D matrix, 2D biofilm, or directly on the polystyrene surface of the 24-well plate (Figure 5A). The peak of TNF-α production was observed in the parasite:cell ratio of 3:1 in the three experimental variables. However, TGF-β production was significantly higher in the 3D matrix model than in the other groups (Figure 5B). Indeed, TGF-β has been related to a mechanism of parasite escape [41,42,43,44,45]. Therefore, this cytokine profile could explain the higher parasite release on the 3D matrix model.

### 3.5. Activated Macrophages Reduced Trypomastigote Release in the 3D Matrix In Vitro

**Figure 6:**
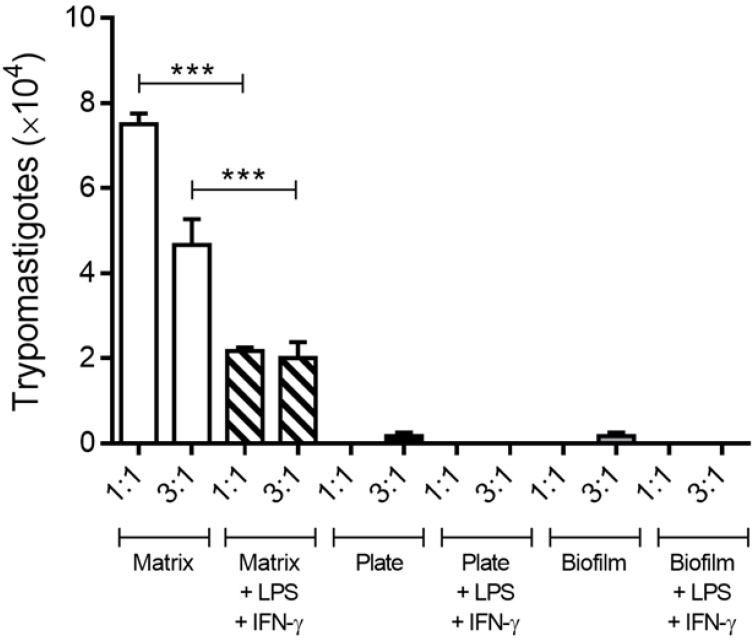
IFN-γ and LPS reduce the trypomastigote release in macrophage culture grown on a 3D matrix. Peritoneal macrophages from BALB/c mice (6 × 10^5^) were cultured on the 3D matrix or directly on the wells of 24-well plates and incubated for 3 h for adhesion at 5% CO_2_ and 37 °C. Cell cultures were infected with metacyclic forms of *T. cruzi* clone Dm28c in proportions of 1:1 (6 × 10^5^) and 3:1 (18 × 10^5^) stimulated with 10 ng/mL lipopolysaccharide (LPS from *Salmonella typhimurium*, Sigma Chemical Co.) and 40 U/mL of IFN-γ for 24 h. After 10 days of infection, the parasites released in the culture supernatants were counted using the Neubauer chamber. The results (means ± SE of triplicates) presented are representative of three experiments performed separately. *** *p* < 0.001.

To verify any changes in the ability of macrophages cultured on the 3D matrix to exert their trypanocide activity in cells pre-treated with IFN-γ and LPS by 24 h and then infected with metacyclic forms of *T. cruzi*, after 10 days, the number of trypomastigotes in the supernatants was evaluated. The presence of IFN-γ and LPS caused a significant reduction in the free trypomastigote forms in the supernatants of macrophage culture grown on a 3D matrix and no similar effect on 2D biofilm and plate cultures (Figure 6). Nevertheless, in macrophages, stimulation with IFN-γ, LPS, or both promotes the production of large amounts of nitric oxide (NO) [46], and the trypanocide activity of macrophages is mainly mediated by NO production. However, we did not detect any nitric oxide in the supernatant of the cultures in our models after infection.

### 3.6. Morphological Alteration of Infected Macrophages Cultured in 3D Matrix Cultures

**Figure 7:**
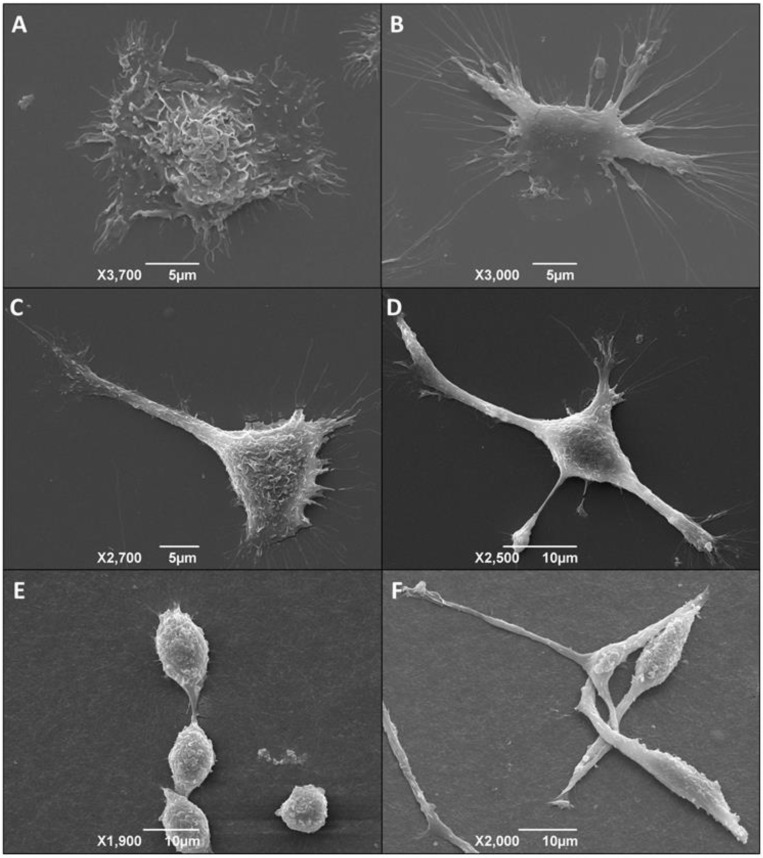
*T. cruzi*-infected macrophages present a more stellar and elongated shape compared to non-infected ones. Morphology and adhesion of non-infected and infected macrophages cultured on a plate, 2D biofilm, and 3D matrix. Scanning electron microscopy of peritoneal macrophages from BALB/c mice (6 × 10^5^/well) cultured directly on polystyrene plate (**A**,**B**), 2D biofilm (**C**,**D**), or 3D matrix (**E**,**F**). The model was divided into non-infected (**A**,**C**,**E**) and infected (**B**,**D**,**F**) groups. For the infected groups, after three hours of adhesion of macrophages, the cultures were infected overnight with metacyclic forms of *T. cruzi* clone Dm28c at a parasite:cell ratio of 1:1. Images were obtained 48 h after infection.

We also used scanning electron microscopy (SEM) to evaluate macrophage morphological changes. Initially, we cultured peritoneal macrophages from normal mice directly on the surface of 24-well plates. Once adhered, we infected the macrophages with metacyclic trypomastigote forms of the *T. cruzi* Dm28c clone or not (control group). Afterward, in order to further explore morphological changes and macrophage adhesion, we performed an SEM analysis of infected and non-infected macrophages cultured directly on plates, 2-D biofilm, and on 3D matrix. The images obtained showed that the non-infected cells grown directly on the polystyrene surface, 2D biofilm, and 3D matrix were rounded and less elongated (Figure 7A,C,E), but *T. cruzi* infected cells were stellar, larger, and shown a more spread pattern (Figure 7B,D,F). These data suggest a direct effect of the parasite infection on macrophage spreading. Infected cells also presented similar extensions to filopodia. However, additional experiments with specific markers are necessary to make sure that these extensions are, in fact, filopodia.

**Figure 8:**
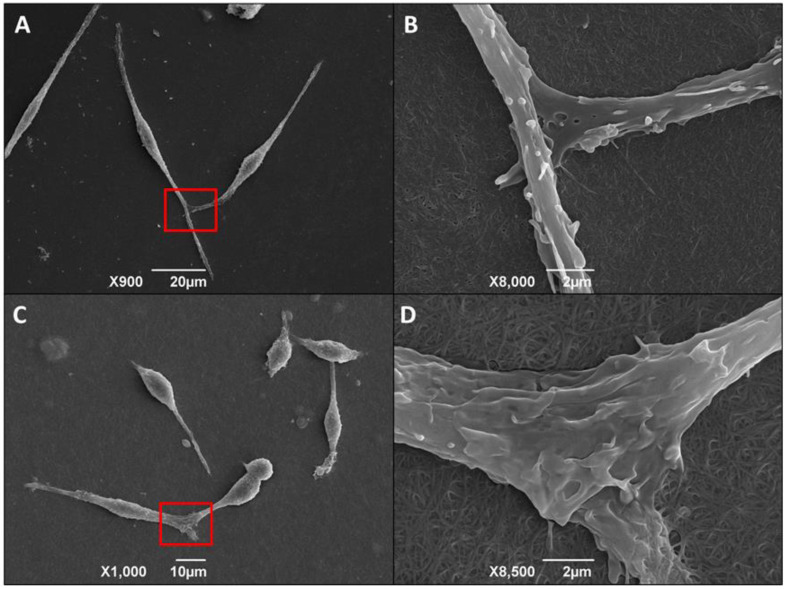
*T. cruzi*-infected macrophages elongate by attaching to the 3D matrix and to each other. Elongated macrophage ties. Adhesion and morphology of macrophages cultured on 3D matrix. Scanning electron microscopy of peritoneal macrophages from BALB/c mice (6 × 10^5^/well) cultured on a 3D matrix. After three hours of adhesion of macrophages to the substrate, cultures were infected overnight with metacyclic forms of clone Dm28c at a parasite:cell ratio of 1:1. Images were obtained 48 h after infection. In the detail of *T. cruzi* figures (**A**,**C**), the contact between the cells in (**B**,**D**) is in greater magnitude. Red rectangles in (**A**,**C**) indicate maximized figures (**B**,**D**), respectively.

During the SEM analysis, we also observed the cell-cell contacts, with overlapping of extensions and their anchoring on the 3D matrix (details in red) (Figure 8).

**Figure 9:**
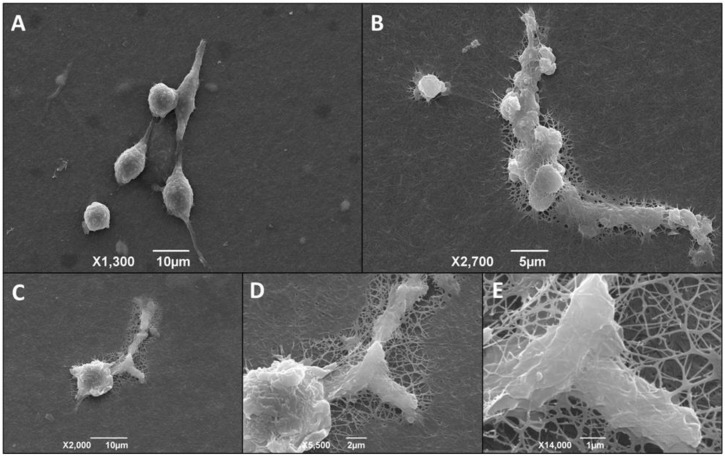
*T cruzi*-infected macrophages remodel their shape and enter the 3D matrix for displacement. Adhesion and morphology of macrophages cultured on 3D matrix. Scanning electron microscopy of peritoneal macrophages from BALB/c mice (6 × 10^5^/well) cultured on a 3D matrix. Representative images of the non-infected group (**A**) and infected group (**B**). For the infected groups, after three hours of adhesion of macrophages, the cultures were infected overnight with metacyclic forms of *T. cruzi* clone Dm28c at a parasite:cell ratio of 1:1. The images were obtained 48 h after infection, and we observed migratory macrophages remodeling the mesh for its displacement (**C**) 2000× magnification. (**D**) 5500× magnification. (**E**) 14,000× magnification.

We also observed that the infected macrophages are “plugging” and remodeling the collagen type I mesh for displacement (Figure 9). It is important to note that these cells present a profile of migratory macrophages. This phenomenon was observed only in cultures of infected macrophages cultured on a 3D matrix.

## 4. Discussion

The use of the monolayer cell culture enabled a better understanding of the molecular mechanisms of the cell, allowing important scientific advances in the different areas of research. However, responses obtained in monolayer cell culture are not always completely satisfactory since the behavior of the cells always differs from the ones observed in vivo, considering the lack of cell-matrix interactions in these types of protocols. Three-dimensional (3D) cell culture was initially derived from cell culture in monolayers. The differential of the 3D culture allows the cells to explore the three dimensions of space, thus enhancing the interaction between cells and the environment. Therefore, the use of 3D models composed of synthetic polymers or natural materials, such as collagen, laminin, and fibronectin, among others, as physical support lets us mimic the spatial conditions to which cells are subjected in vivo [19,47,48]. The use of cell cultures in 3D models has led to infection studies with bacteria and viruses, reflecting the natural infection process. These models may be adapted for the study of other infectious agents, such as parasites [49,50,51].

It is well known across the literature that the invasion process of host cells by *T. cruzi* takes at least 10 min [52], then it takes about 1 h for lysosomal markers to reach the vacuoles containing the parasite [53]. It is during this period that the membrane fragmentation of the parasitoid vacuole and consequent release of the parasites into the cytoplasm occurs [52]. Once in the cytoplasm, the amastigote forms remain quiescent from 24 to 44 h, after which the division process begins. The generation time of the amastigotes can vary within the 8 to 15 h range, depending on the strain, while the cytokinesis takes between 20 and 30 min [54]. Cell rupture is necessary for the process of transformation of the amastigote forms into trypomastigotes and takes around 2 to 5 days [23].

In an intriguing way, it was verified in our study that after 96 h post-infection, it was possible to quantify trypomastigote forms in the supernatants of infected macrophages with or without the 3D matrix. One hypothesis to explain the replication and early release of trypomastigote forms in macrophages cultured on the collagen type I substrate is that contact of the host cell with the template modulates the activity of key host molecules such as surface receptors, cytoskeleton proteins or proteins cell signaling [55]. This has crucial implications regarding the infectivity of *T. cruzi* since in our 3D matrix model, the production of TGF-β was much higher compared to other 2D models, suggesting a possible effect of the 3D matrix in macrophage modulation towards a more regulatory and repairing profile, as in the case of alternatively activated macrophages (M2) [15]. Interestingly, M2 macrophages display altered isoforms of fibronectin [56], and this may influence the host-parasite interaction, as previously described for *T. cruzi* [57]. Therefore, the observed migratory macrophages could have their components of interaction with the matrix altered by TGF-β. Similar behavior has already been described in in vitro cultures with macrophages in collagen I matrices, in which the cells presented a lower release of inflammatory cytokines such as IL-6 and TNF-α and greater release of the regulatory cytokine IL-10 [58]. Therefore, our model presents similar characteristics in the modulation of macrophages in the collagen matrix, showing a greater presence of TGF-β. However, it is worth mentioning that this phenomenon was exclusively conditioned by infection with *T. cruzi* in our model. Dealing more specifically with the possible relationships between the infection and the production of TGF-β, it is well documented that the parasite can adhere to cells genetically deficient in TGF-β receptors TβRI or TβRII but is unable to penetrate and replicate within these cells, as opposed to within competent cells. The infection in the receptor mutants is restored when the TGF-β receptor genes are transfected [59]. *T. cruzi* can also directly trigger the signaling pathway required for entry into the host cell using a factor similar to TGF-β, detectable in extracts of the parasite in the infective form [59]. Furthermore, the anti-TGF-β neutralizing antibody inhibits the invasion of *T. cruzi* in cardiomyocytes [60], and the pre-treatment of host cells with the TGF-β receptor inhibitor, SB-431542, also can lead to a similar invasion inhibition [61]. The data suggest that, in a complete scenario, the parasite needs to activate latent TGF-β, which is associated with the extracellular matrix components, to invade host cells. Probably, TGF-β would be activated by proteases [62] and sialidases [59] secreted by the parasite, capable of processing the latent form into an active peptide.

Furthermore, immunity elicited by TGF-β is particularly significant in the immunopathology of Chagas disease, being associated with regulatory mechanisms of fibrosis induction by *T. cruzi* infection [63]. In addition, increased TGF-β signaling is associated with greater adherence, internalization, and recognition of the parasite by host cells, including greater TGF-β receptor expression, which also increases affinity for the parasite [64,65]. Although we were not able to evaluate the expression of this receptor in our model, our data suggest that the presence of a cellular matrix is crucial for the immunopathogenesis of *T. cruzi,* as seen by the increased replication on the 3D matrix, and therefore, in vitro models that mimic this structural component tend to approximate the actual modulation of macrophages that occurs physiologically.

The trypomastigote forms when phagocytosed by macrophages differ from amastigote forms, with the macrophage state being the determinant for their survival. IFN-γ-activated macrophages are able to partially destroy the parasites [44,45,66,67]. The results obtained in our study corroborate the previous studies since the presence of collagen I did not alter the activation of the macrophages by IFN-γ and LPS, which still were able to control the intracellular replication of *T. cruzi* [68].

The establishment of *T. cruzi* infection depends on a series of events involving interactions between parasite and host molecules [69]. This process has been extensively studied in in vitro models, with mammalian cell lines or peritoneal macrophages residing in adhered monolayers on rigid surfaces, such as, for example, cell culture polystyrene plates (2D model) [70].

In the present study, we decided to evaluate the morphological alterations of resident peritoneal macrophages infected by *T. cruzi* and the replication of the parasite in cells cultured on a 3D matrix in the 3D model. It is important to highlight the uniqueness and importance of our study since no other similar study has ever been developed.

The 3D matrix induces a reorganization of the actin cytoskeleton of the infected macrophages [71,72], which would favor the formation of the observed projections and consequently facilitate the infection of the cells.

The use of SEM shows that macrophages cultured and infected by *T. cruzi* on the 3D matrix were scattered, with fine extensions and increased cell-to-cell contact. In addition, we also found that the extensions were anchored in the 3D matrix, allowing macrophage migration through the collagen type I substrate [73]. We did not observe the same morphological changes in macrophage monolayers cultured on 24 plate wells or when cells were cultured having the 2D biofilm as the substrate.

Macrophage culture on the 3D matrix favored the formation of migratory macrophages [74]. This phenomenon was observed only in macrophages infected by *T. cruzi* in our study. Extracellular matrices create physical barriers to cell migration. In order to reach the different tissue sites, macrophages carry out transmigration through the endothelial wall and through basolateral membranes and interstitial spaces [74,75]. Although migrating macrophages in two dimensions have been extensively studied, the mechanism involving migration in 3D models remains unclear.

## 5. Conclusions

This study demonstrates for the first time the higher replication of *T. cruzi* when using the 3D matrix during macrophage infection in vitro and the presence of migratory macrophages in a model of parasite infection. Because they present a larger migratory capacity in the 3D matrix, these cells may be involved in cellular signaling or favoring the exacerbation of the immune response in situ [76].

The physical support provided by the 3D matrix layer used in our system can provide relevant information to a better understanding of the *T. cruzi* invasion process and why the parasitic replication is enhanced when the 3D matrix acts as a scaffold for infected cells, where several parasites and host cell molecules can interact and activate signaling pathways, promoting parasite internalization. In addition, this study provides what may become an important model for the understanding of the immunomodulatory mechanisms of infections and even for initial drug screening panels.

## Data Availability

The data is contained within this article.
